# MulCNN: An efficient and accurate deep learning method based on gene embedding for cell type identification in single-cell RNA-seq data

**DOI:** 10.3389/fgene.2023.1179859

**Published:** 2023-04-04

**Authors:** Linfang Jiao, Yongqi Ren, Lulu Wang, Changnan Gao, Shuang Wang, Tao Song

**Affiliations:** ^1^ College of Computer Science and Technology, China University of Petroleum, Qingdao, China; ^2^ Department of Artificial Intelligence, Faculty of Computer Science, Polytechnical University of Madrid, Madrid, Spain

**Keywords:** single-cell sequencing, scRNA-seq, cell type identification, convolutional neural Networks, gene expression feature extraction

## Abstract

Advancements in single-cell sequencing research have revolutionized our understanding of cellular heterogeneity and functional diversity through the analysis of single-cell transcriptomes and genomes. A crucial step in single-cell RNA sequencing (scRNA-seq) analysis is identifying cell types. However, scRNA-seq data are often high dimensional and sparse, and manual cell type identification can be time-consuming, subjective, and lack reproducibility. Consequently, analyzing scRNA-seq data remains a computational challenge. With the increasing availability of well-annotated scRNA-seq datasets, advanced methods are emerging to aid in cell type identification by leveraging this information. Deep learning neural networks have great potential for analyzing single-cell data. This paper proposes MulCNN, a multi-level convolutional neural network that uses a unique cell type-specific gene expression feature extraction method. This method extracts critical features through multi-scale convolution while filtering noise. Extensive testing using datasets from various species and comparisons with popular classification methods show that MulCNN has outstanding performance and offers a new and scalable direction for scRNA-seq analysis.

## 1 Introduction

Single-cell transcriptomics technologies hold significant potential for advancing our understanding of cellular heterogeneity in complex tissues (Luecken and Theis, 2019; Longo et al., 2021). Among these technologies, single-cell RNA sequencing (scRNA-seq) has become a central tool for identifying and characterizing cell types, states, and lineages in diverse biological contexts (Andrews and Martin, 2018; Zhang et al., 2021a; Xu et al., 2023). It enables analysis of the transcriptome of individual cells, thereby transforming biological research and enabling the classification of cell types across multiple species, tissues, and contexts (Zhang et al., 2021b; Karlsson et al., 2021). However, scRNA-seq experiments often generate vast amounts of data, and large projects like the Human Cell Atlas may involve thousands to millions of cells (Adlung and Amit, 2018). Thus, fast and efficient computational methods are essential for scRNA-seq analysis.

Clustering and cell type identification are crucial steps in single-cell RNA sequencing analysis, with the identification of cell types being particularly important for revealing cellular heterogeneity in different tissues, developmental stages, and organisms (Kiselev et al., 2010; Philpott et al., 2021). This knowledge can enhance our understanding of cellular and genetic functions in both health and disease contexts (Cohen et al., 2021). However, despite the advanced capabilities of scRNA-seq, its high dimensionality, sparsity, and technical noise pose significant challenges to cell type identification (Yuan and Kelley, 2022). Furthermore, identifying cell populations in large datasets presents even greater difficulties, as many existing scRNA-seq clustering methods are unable to handle such datasets at scale.

Popular unsupervised clustering methods for inferring cell types from scRNA-seq data typically involve two steps. First, an unsupervised algorithm is used to cluster cells based on their gene expression profiles. Second, marker genes that are uniquely and highly expressed within each cluster are used to assign cell types (Petegrosso et al., 2020). However, using canonical markers for cell type annotation requires extensive background knowledge and may not always be reliable. Some new cell types may lack known markers, while some canonical markers may be expressed by multiple cell types. Moreover, several sources of variation can influence cluster formation, including those that are not directly related to cell type (Kiselev et al., 2010). Consequently, setting appropriate clustering parameters and assigning identities to cells in each cluster are critical steps. The popular unsupervised scRNA-seq clustering methods, such as Louvain (Blondel et al., 2008), DESC (Li et al., 2020), and SAVER-X (Wang et al., 2019), are widely used, but they have limitations. For instance, these methods do not take advantage of cell type-specific gene expression information and perform poorly in datasets containing batch processing.

Automated cell type identification methods aim to identify commonalities between scRNA-seq datasets and address the inherent noise and variability of the data (Tang et al., 2009). In fact, scRNA-seq datasets are affected by several confounding factors, including the sequencing platform, sequencing depth, and sample preparation process. The multidimensional nature of scRNA-seq data and the presence of noise make machine learning methods highly useful for various tasks in the analytics pipeline, such as dimensionality reduction (Becht et al., 2019). Supervised cell classification using labeled reference data is gaining popularity over unsupervised clustering algorithms as more scRNA-seq data becomes available. This approach, which involves using machine learning techniques for supervised classification, represents a classic example of supervised classification in machine learning (Amodio et al., 2019).

Current automatic cell classification methods fall into three categories. The first relies on information from publicly available databases and ontologies describing cell type-specific markers. The second approach uses labeled scRNA-seq datasets as input for cell type identification to find the best correlation between reference and query datasets, such as scmap and Seurat 3.0 (Kiselev et al., 2017; Stuart and Satija, 2019; Pasquini et al., 2021). The third and currently popular approach is supervised learning, which involves training a classifier with a labeled reference dataset (Eraslan et al., 2019). Popular supervised learning algorithms include those based on the support vector machine (SVM) method, such as Moana and scPred (Wagner and Yanai, 2018; Alquicira-Hernandez et al., 2019). ItClust is a machine learning method based on supervised pre-trained transfer learning (Hu et al., 2020), while ACTINN and scVI are supervised classification methods based on neural networks (Romain et al., 2018; Ma and Pellegrini, 2020). Neural networks are popular in the biomedical field due to their powerful ability to resolve non-linear relationships between categories and features, as well as recent advances in computational speed (Wainberg et al., 2018). However, existing supervised methods rely heavily on the quality of the training data and often have poor accuracy in classifying cell types that are not present in the training data. Recent studies have shown that deep learning has good performance when applied to image and text datasets (Xie et al., 2016; Guo et al., 2017). Additionally, traditional clustering methods perform poorly in high dimensions due to the “curse of dimensionality,” while deep learning methods can convert high-dimensional raw scRNA-seq data into low-dimensional representations (Lin et al., 2017; Li et al., 2020).

Therefore, we propose a deep learning method for cell classification called MulCNN. This method is based on a multi-level convolutional neural network that utilizes a multi-scale convolutional pooling operation, incorporating principal component analysis to extract multidimensional features to train the model to predict cell types. Extensive evaluation using data from different species and tissues generated by various scRNA-seq schemes demonstrates that MulCNN considerably enhances the accuracy of cell type classification compared to popular unsupervised clustering and supervised cell type classification algorithms.

## 2 Materials and methods

### 2.1 Dataset

This paper presents an analysis of four publicly available scRNA-seq datasets generated using InDrop [Baron et al. (2016) data], SmartSeq2 [Segerstolpe et al. (2016) data], Fluidigm C1 [Lawlor et al. (2017) data], and SMARTer [Xin et al. (2016) data]. The dataset details are summarized in Table 1.

**TABLE 1:** Datasets analyzed in this paper.

Dateset	Species	Number of genes	Number of cells	Number of types	Platform
Baron_Human [22]	Human	20,125	8,569	acinar(958); activated_stellate(284); alpha(2,326); beta(2,525); delta(601); ductal(1,077); endothelial(252); epsilon(18); gamma(255); macrophage(55); mast(25); quiescent_stellate(173); schwann(13); t_cell(7)	InDrop
Baron_Mouse [22]	Mouse	14,878	1886	activated_stellate(14); alpha(191); B_cell(10); beta(894); delta(218); ductal(275); endothelial(139); gamma(41); immune_other(8); macrophage(36); quiescent_stellate(47); schwann(6); T_cell(7)	InDrop
Segerstolpe [23]	Human	26,178	2068	acinar(185); alpha(886); beta(270); delta(114); ductal(386); endothelial(16); epsilon(7); gamma(197); mast(7)	SMART-Seq2
Lawlor [24]	Human	26,616	617	Acinar(24); Alpha(239); Beta(264); Delta(25); Ductal(28); Gamma/PP(18); Stellate(19)	Fluidigm C1
Xin [25]	Human	39,851	1,600	alpha(946); beta(503); delta(58); PP(93)	SMARTer

To normalize the data, we applied a uniform processing pipeline to all datasets. Specifically, we discarded genes with less than 200 non-zero values. We then performed cell-level normalization, where the UMI count for each gene in each cell was divided by the total number of UMIs in the cell, multiplied by 10,000, and transformed using the natural log function. Finally, we randomly split the data into training (70%), validation (15%), and test (15%) sets.

Our approach ensures that the datasets are pre-processed consistently, which allows for a fair comparison of performance between the different algorithms.

### 2.2 Model architecture

**FIGURE 1:**
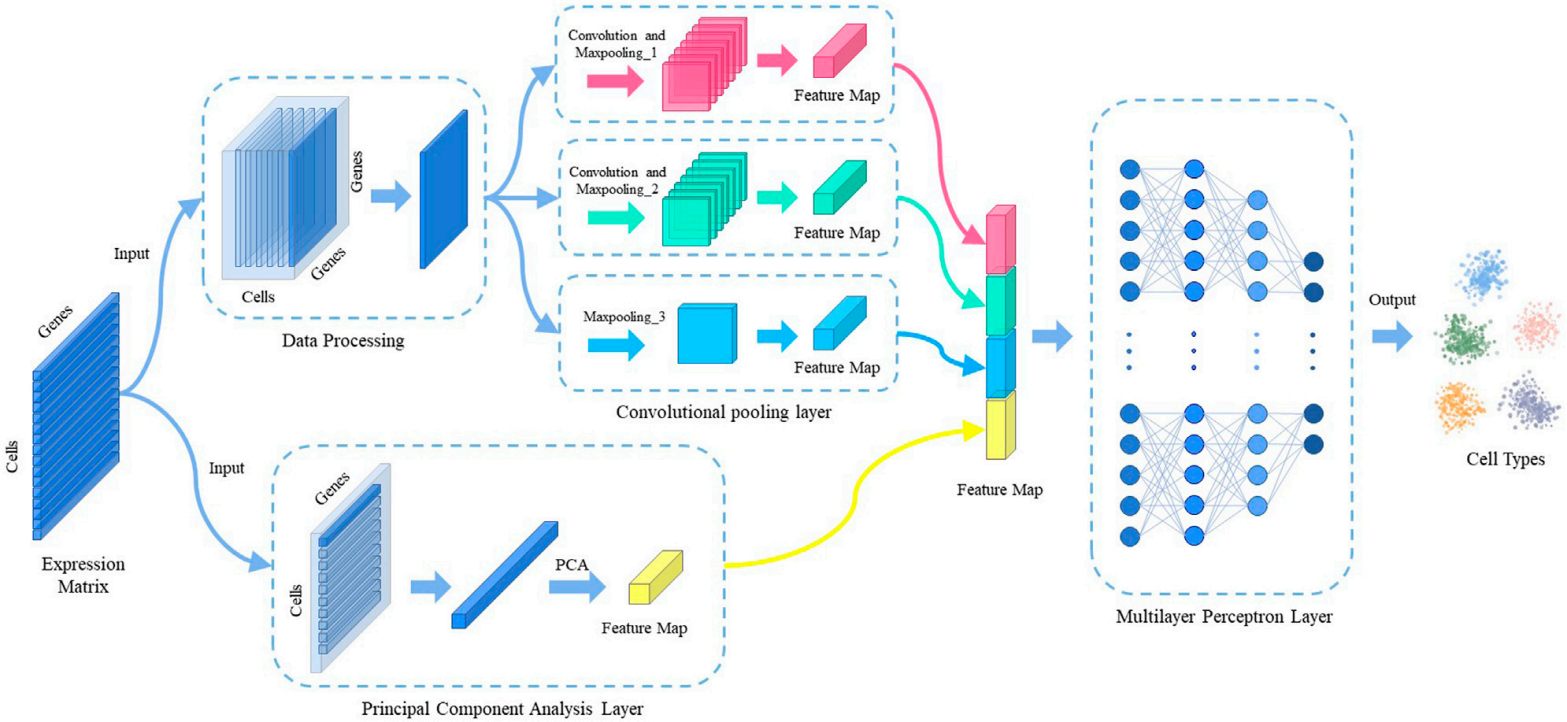
The overall architecture of MulCNN.

The model consists of three main components: data processing, feature extraction, and predictive classification. Figure 1 shows the overall architecture of the model. The gene expression matrix data are first normalized by counts per million (CPM) and then subjected to multi-scale convolutional pooling and principal component analysis (PCA) to extract cell-type-specific gene expression features. Prior to convolutional pooling, each gene expression data line is transformed into a two-dimensional matrix representation. Finally, a multilayer perceptron layer is applied for predictive classification.

### 2.3 Convolutional pooling layer

**FIGURE 2:**
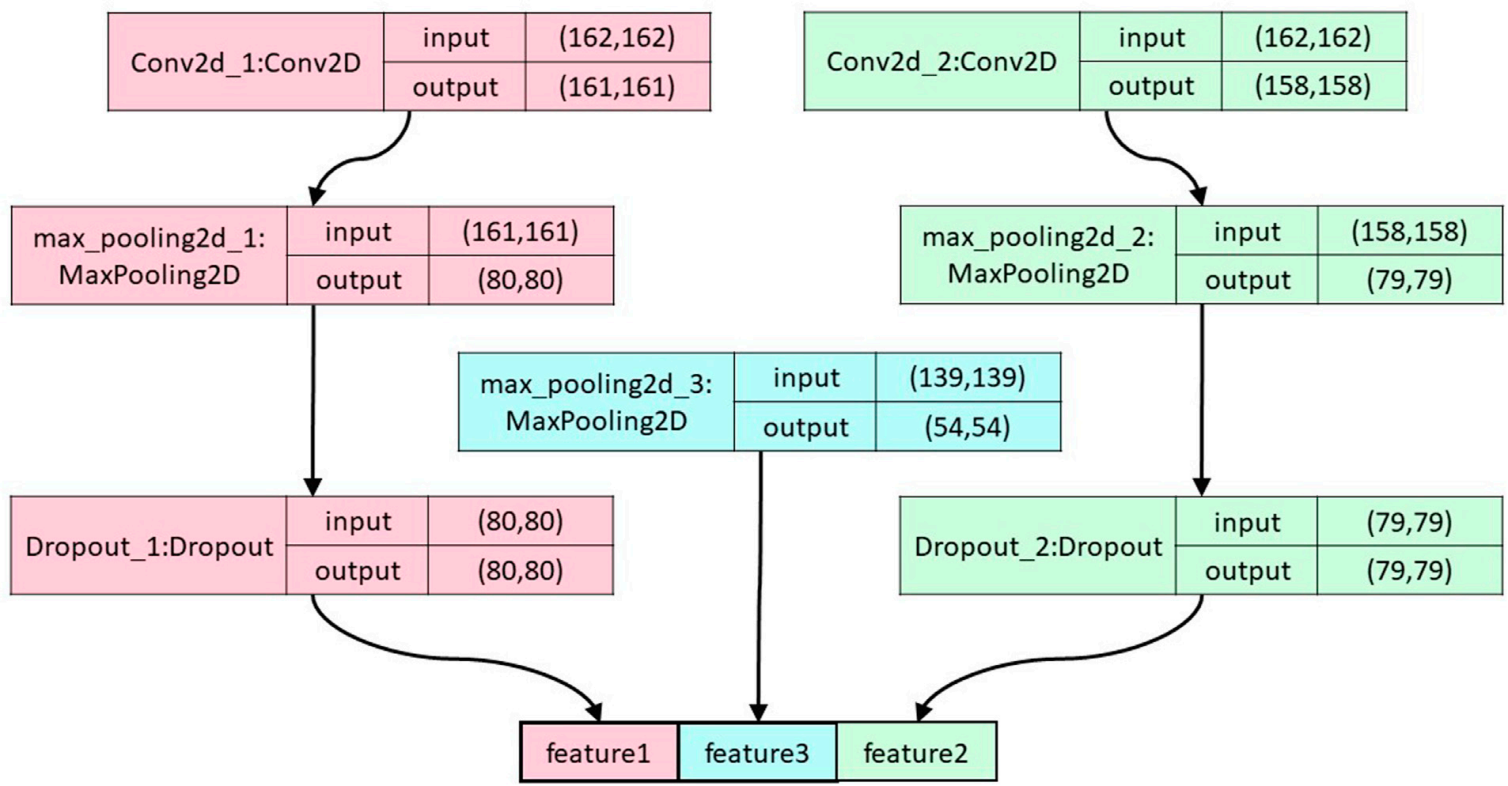
The overall structure of the convolutional pooling layer. The figure shows the segerstolpe dataset as an example.

To avoid overfitting, the number of convolutional pooling layers in the neural network is limited to three (Szegedy et al., 2015). The topology of this module is illustrated in Figure 2. The formula for convolution is expressed as follows:

$$S\left(i,j\right)=\left(I*W\right)\left(i,j\right)=\underset{m}{\sum }\underset{n}{\sum }I\left(i+m,j+n\right)W\left(m,n\right).$$

Where I is the two-dimensional input, W is the convolution kernel, and the result $S\left(i,j\right)$ is the feature mapping.

As the activation function, we use ReLU, defined as follows:

$$ReLU\left(x\right)=\max\left(0,x\right).$$

Where x is the linear operations returned by the current layer.

### 2.4 Principal component analysis layer

The Principal Component Analysis (PCA) layer uses PCA dimensionality reduction as its primary algorithm. PCA is a widely-used linear dimensionality reduction method that aims to map high-dimensional data into a lower-dimensional space using linear projection, while retaining the maximum variance (i.e., most information) of the data in the projected dimensions. By reducing the number of dimensions in the data, PCA enables the use of fewer dimensions while preserving most of the original data points’ characteristics.

Due to the high dimensionality and sparsity of scRNA-seq data, we downscaled the gene expression data to minimize the loss of information when extracting effective cell-type-specific features. Specifically, we projected the original features onto the dimension with the most projected information. After dimensionality reduction, projecting the original features onto these dimensions results in less information loss, allowing us to extract cell-type-specific gene expression features that are more beneficial to our model.

### 2.5 Multilayer perceptron layer

The cell type-specific gene expression features extracted from the convolutional pooling layer are combined with the features extracted from the principal component analysis layer and fed to the multilayer perceptron layer. This neural network consists of one input layer, three hidden layers, and one output layer. The number of nodes in the input layer is the same as the number of features extracted using the convolutional pooling and PCA. The hidden layers have 128, 128, and 64 nodes, respectively. The number of nodes in the output layer is equal to the number of cell types in the dataset.

Forward propagation is implemented as follows:

$$x^{\left[i\right]}=g\left(W^{\left[i\right]}x^{\left[i-1\right]}+b^{\left[i\right]}\right).$$

Where $x^{\left[i\right]}$ is the output of the i th layer ( $x^{\left[0\right]}$ indicates the input layer), $b^{\left[i\right]}$ is the bias of the i th layer, $W^{\left[i\right]}$ is the weight matrix of the i th layer and g is the activation function.

The ReLU function is used as the activation function for the input and hidden layers. The softmax function was used for the output layer, which is defined as:

$$softmax\left(x_{\left[j\right]}\right)=\frac{\exp\left(x_{\left[j\right]}\right)}{\sum_{j=1}^{k}\exp\left(x_{j}\right)}$$

Where $x_{\left[j\right]}$ is the j th element of the input vector for the output layer, which has k elements, representing a total of k cell types in the training set.

### 2.6 Loss function and parameters setting

The cross-entropy function is used as the loss function in our model, which is defined as:

$$J\left(W,b\right)=-\frac{1}{n}\sum_{i}^{n}\left[y_{i}\log{\hat{y}}_{i}+\left(1-y_{i}\right)\log\left(1-{\hat{y}}_{i}\right)\right].$$

Where vector $y_{i}$ is the true label for the cell, vector ${\hat{y}}_{i}$ is the predicted label for the cell, i is the sample and n is the total number of samples.

The learning rate is set to 0.0001, and we use the SGD optimization model with the model parameters shown in Table 2.

**TABLE 2:** The values of parameters used in our model.

Parameters		Range
Conv2d_1	Number of filters	256
	Kernel size	(2,2)
	Dropout	0.25
	Activation	ReLU
Conv2d_2	Number of filters	128
	Kernel size	(5,5)
	Dropout	0.25
	Activation	ReLU
max_pooling2d_1		(2,2)
max_pooling2d_2		(2,2)
max_pooling2d_3		(3,3)
Optimizer		SGD
Learning rate		0.0001
Epoch		300
Batch size		32

The neural network model is implemented using TensorFlow 2.4.0 and written in Python 3.6. The model uses the CategoricalCrossentropy loss function and is initialized with a seed to ensure reproducibility. The learning rate is set to 0.0001, and the network is trained for 300 epochs with a batch size of 32 samples per global step. Dropout regularization with a parameter of 0.25 is also employed to prevent overfitting.

## 3 Results

### 3.1 Evaluation metrics

In order to showcase the scalability and advantages of MulCNN, we conducted analyses on several single-cell RNA sequencing datasets from different species, generated using various platforms. Table 3 displays the evaluation metrics we employed.

**TABLE 3:** The metrics used in the evaluation of model.

	Actual positive	Actual negative
Predicted Positive	TP	FP
Predicted Negative	FN	TN
True Positive Rate (TPR)	TP/(TP + FN)	
False Positive Rate (FPR)	FP/(FP + TN)	
Precision	TP/(TP + FP)	
Recall	TP/(TP + FN)	
Accuracy	(TP + TN)/(TP + FP + FN + TN)	
AUC	$AUC=\frac{1}{2}\sum_{i-1}^{m-1}\left(x_{i+1}-x_{i}\right)\left(y_{i}+y_{i+1}\right)\left(x:FPR,y:TPR\right)$	
F_1-score_	$F_{1-score}=2\times \frac{Precision\times Recall}{Precision+Recall}$	

### 3.2 Comparison with other cell type identification methods

#### 3.2.1 Comparison with unsupervised clustering methods

To compare the effectiveness of MulCNN, we evaluated its performance against three unsupervised clustering methods and six supervised classification methods. Specifically, we compared MulCNN against three unsupervised methods: Louvain, DESC, and SAVER-X + Louvain. Louvain is a clustering method proposed by Blondel et al. (2008) that relies on the degree of community module metric. Li et al. (2020) proposed DESC, an unsupervised deep embedding method that iteratively improves a clustering objective function to cluster scRNA-seq data. Additionally, Wang et al. (2019) presented SAVER-X, a neural network-based transfer learning algorithm originally designed to denoise gene expression. SAVER-X collects gene expression characteristics from a source dataset, then denoises the target data’s unique molecular identifier counts using previously learned gene expression information. As these methods are unsupervised clustering techniques, they do not use any labeling information from the dataset to identify cell types.

We conducted experiments on the Lawlor and Segerstolpe datasets obtained from Fluidigm C1 and SMART-Seq2, respectively. We first evaluated the performance of MulCNN on each dataset individually, and then combined the two datasets to test its ability to classify the data in the presence of batch effects. Although both Lawlor and Segerstolpe are derived from the human pancreas, the fact that they were processed and measured on different platforms introduces technical biases that do not correlate with the biological state. This makes cell classification challenging. To integrate the two datasets, we used an expression value matrix that preserves the intersection of gene features.

The performance of the Louvain and DESC algorithms depends on the resolution, a hyperparameter that determines the number of clusters and must be provided by the user. We chose a resolution range of 0.2–2, in steps of 0.2. To compare the performance of different clustering techniques, we used the Adjusted Rand Index (ARI). The ARI measures the degree of similarity between clustering labels generated by a clustering method and reference cluster labels. The formula for calculating ARI is shown below:

$$\begin{array}{c} ARI=\frac{\underset{jj^{\prime }}{\sum }\left(\begin{array}{l} n_{jj^{\prime }}\\ 2 \end{array}\right)-\frac{\left[\underset{j}{\sum }\left(\begin{array}{l} a_{j}\\ 2 \end{array}\right)\underset{j^{\prime }}{\sum }\left(\begin{array}{l} b_{j^{\prime }}\\ 2 \end{array}\right)\right]}{\left(\begin{array}{l} n_{jj^{\prime }}\\ 2 \end{array}\right)}}{\frac{1}{2}\left[\underset{j}{\sum }\left(\begin{array}{l} a_{j}\\ 2 \end{array}\right)+\underset{j^{\prime }}{\sum }\left(\begin{array}{l} b_{j^{\prime }}\\ 2 \end{array}\right)\right]-\frac{\left[\underset{j}{\sum }\left(\begin{array}{l} a_{j}\\ 2 \end{array}\right)\underset{j}{\sum }\left(\begin{array}{l} b_{j^{\prime }}\\ 2 \end{array}\right)\right]}{\left(\begin{array}{l} n_{jj^{\prime }}\\ 2 \end{array}\right)}} \end{array}$$ (1)

Where $n_{jj^{\prime }}$ denotes the number of cells assigned to cluster *j* based on reference cluster labels and cluster $j^{\prime }$ based on clustering labels obtained from a clustering algorithm, $a_{j}$ denotes the number of cells assigned to cluster *j* in the reference set, and $b_{j^{\prime }}$ denotes the number of cells assigned to cluster $j^{\prime }$ by the clustering algorithm.

**FIGURE 3:**
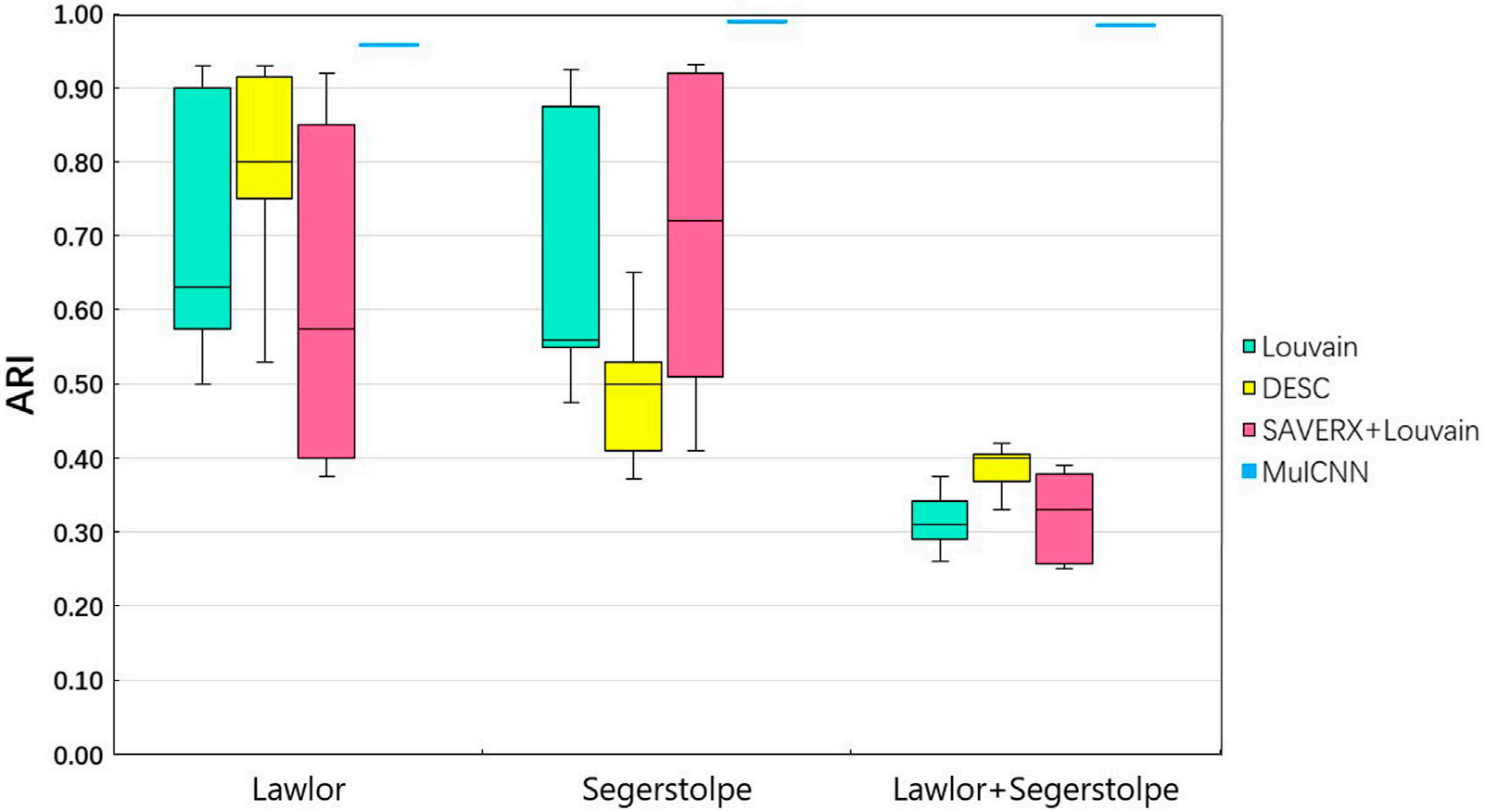
ARI analysis of MulCNN compared with other clustering algorithms.

As shown in Figure 3 [part of the experimental results were obtained from Jian Hu et al. (Alquicira-Hernandez et al., 2019)], the ARI values of Louvain, DESC, and SAVER-X + Louvain vary significantly on the two separate datasets as the resolution parameter changes. In contrast, MulCNN does not require parameter settings, and its ARI is fixed. The results demonstrate that MulCNN consistently outperforms Louvain, DESC, and SAVER-X + Louvain, even when compared to the best resolutions used by these methods.

**FIGURE 4:**
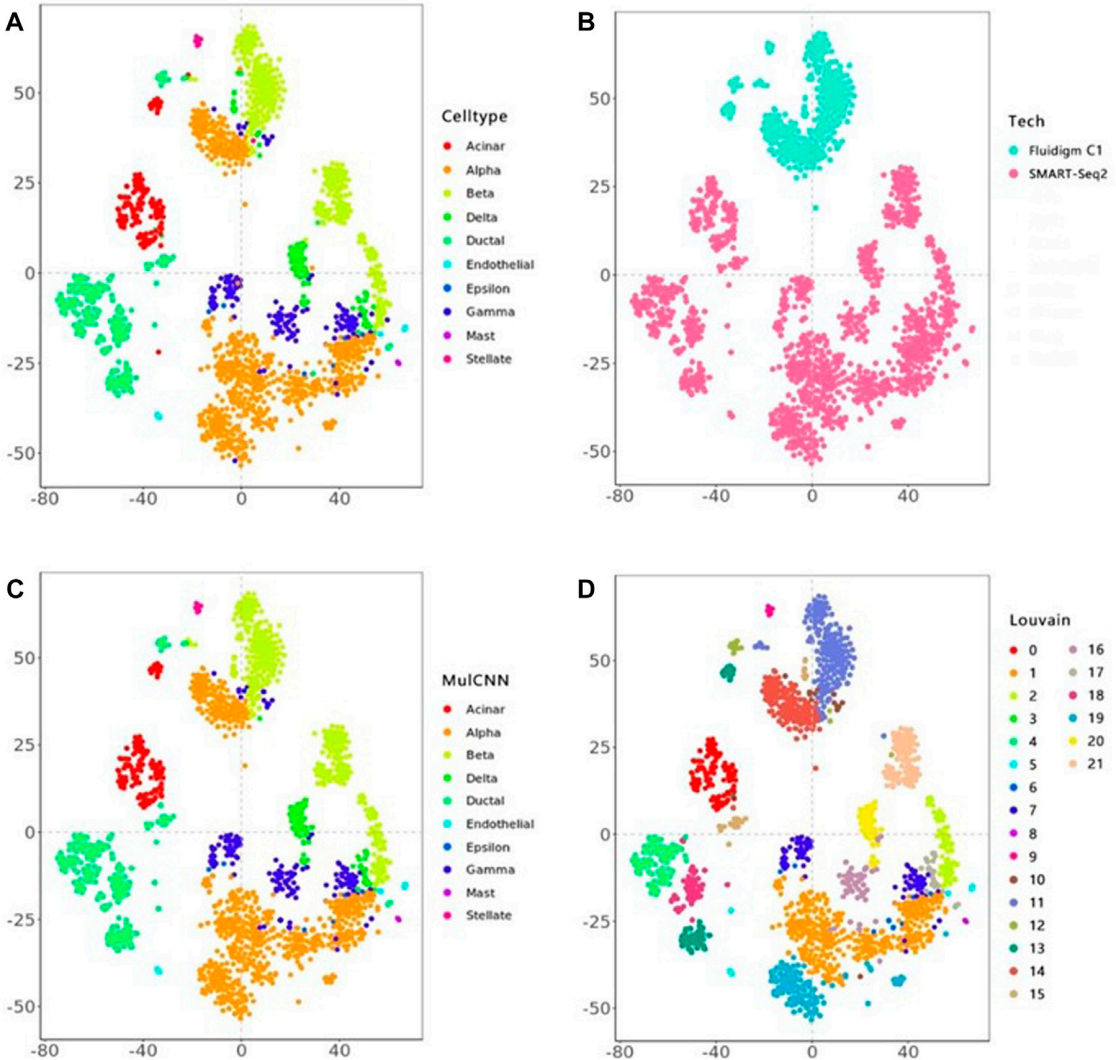
t-SNE visual analysis of the Lawlor + Segerstolpe dataset. **(A)** Distribution of real cell types; **(B)** Distribution of different batches of cells; **(C)** Distribution of cell types predicted by MulCNN; **(D)** Distribution of cell types predicted by Louvain.

Figure 4 visualizes the cell type distribution of the integrated data using t-SNE, in the order of the real cell type distribution of the integrated dataset, the cell distribution from different batches, the cell types distribution predicted by MulCNN, and the cell types distribution predicted by Louvain. As shown in Figure 3, the ARI values for Louvain, DESC, and SAVER-X + Louvain are low, indicating that they tend to cluster cells of the same type but from different datasets into different clusters. With higher resolution, Louvain, DESC, and SAVER-X tend to group major cell types into multiple clusters. In contrast, MulCNN still maintains a high ARI value, outperforming other methods because it can utilize cell type-specific gene expression information in the dataset. MulCNN extracts features for each cell type, avoids extracting features for batch information, and overcomes scRNAseq noise and batch effects generated by different sequencing techniques, thus possessing excellent classification capability. Although SAVER-X denoised the data, it did not make use of the cell type label information in the dataset and therefore was not effective.

#### 3.2.2 Comparison with supervised cell type classification methods

MulCNN was compared to several currently popular supervised methods. However, during actual applications of the model to scRNA data analysis, the labels of the target dataset are often missing. The dataset for which cell types need to be predicted is referred to as the target dataset. In such cases, it is not practical to train the model with most of the target dataset, but there are often many similar and well-labeled datasets that can be used. To better evaluate the performance of MulCNN for practical applications, we pre-trained MulCNN using the Baronhuman dataset, fine-tuned the model with a small portion of the target data, and then used the model to predict the cell types of the target dataset.

We compared MulCNN with several classification algorithms, including scmap, Seurat 3.0, Moana, ItClust, ACTINN, and scVI. Scmap, proposed by Kiselev V Y et al., is a method for annotating cell categories using correlation. It associates each cell in a query dataset with a reference set of cell types or clusters with annotations, using a projection method to identify the best matching cell type or individual cell in the reference dataset (Pasquini et al., 2021). Seurat 3.0 finds anchor cell pairings between well-labeled source datasets and unlabeled target datasets to classify cells in target data (Luecken and Theis, 2019). Wagner F et al. introduced Moana, a hierarchical machine learning framework for constructing robust cell type classifiers from diverse scRNA-Seq datasets. Moana uses a kNN smoothing step to reduce unnecessary noise instead of picking features (Stuart and Satija, 2019). Hu et al. (2020) presented It-Clust, a transfer learning algorithm that incorporates principles from supervised cell type classification methods but additionally uses target data information to ensure sensitivity in classifying cells that are only present in the target data. The ACTINN model uses a neural network with three hidden layers to train the model on a dataset containing specified cell kinds to predict the cell types (Ma and Pellegrini, 2020). Based on hierarchical Bayesian and deep learning, Lopez R et al. suggested scVI as a scalable multitasking tool for learning low-dimensional representations and evaluating scRNA-seq data (Romain et al., 2018).

**FIGURE 5:**
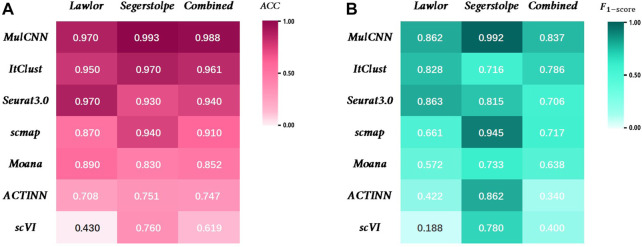
Performance comparison of MulCNN and other supervised algorithms. **(A)** Accuracy comparison on the datasets Lawlor, Segerstolpe,Combined. **(B)** F_1-score_ comparison on the datasets Lawlor, Segerstolpe,Combined.

As shown in Figure 5, MulCNN consistently achieves the highest cell type classification accuracy (ACC) across different datasets, and the F_1-score_ also performs well in general. The formula for the ACC can be expressed as:

$$\begin{array}{c} ACC=\frac{TP+TN}{TP+FP+FN+TN} \end{array}$$ (2)

Where TP is the number of positive instances that were correctly identified by the model as positive, FP is the number of negative instances that were incorrectly identified by the model as positive, FN is the number of positive instances that were incorrectly identified by the model as negative and TN is the number of negative instances that were correctly identified by the model as negative.

The formula for calculating the F_1-score_ is expressed as:

$$\begin{array}{c} F_{1-score}=2\times \frac{Precision\times Recall}{Precision+Recall} \end{array}$$ (3)

Where Precision is the proportion of correctly predicted positive instances out of all instances predicted as positive, Recall is the proportion of correctly predicted positive instances out of all actual positive instances.

**FIGURE 6:**
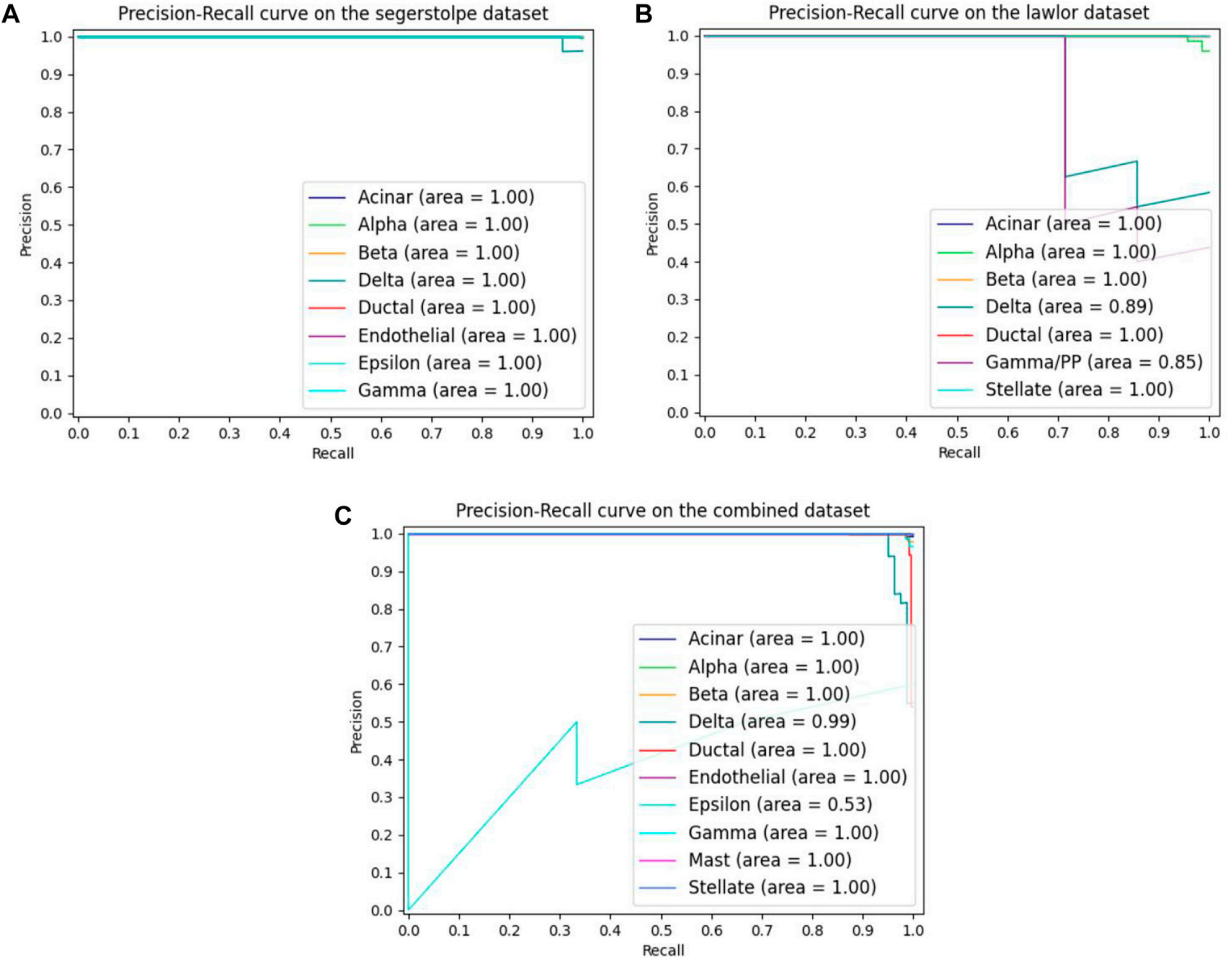
The MulCNN’s precision recall curves for each cell type on different datasets. **(A)** Precision recall curves on the segerstolpe dataset. **(B)** Precision recall curves on the lawlor dataset. **(C)** Precision recall curves on the combined dataset.

As the single-cell transcriptome dataset is usually unbalanced, we plotted the PRC curves on different datasets in order to investigate the classification performance of MulCNN on each cell type, as shown in Figure 6. It can be seen that MulCNN performs particularly well on each cell type in the Segerstolpe and Lawlor datasets. However, on the Combined dataset, the classification performance of Epsilon cell type is poor. By exploring the reasons for this, we found that there were only 7 cell samples of Epsilon type in the dataset. The lack of training samples caused MulCNN to not learn enough and failed to extract effective features, resulting in poor classification results. We will address this issue by collecting more comprehensive and high-quality cell samples.

**FIGURE 7:**
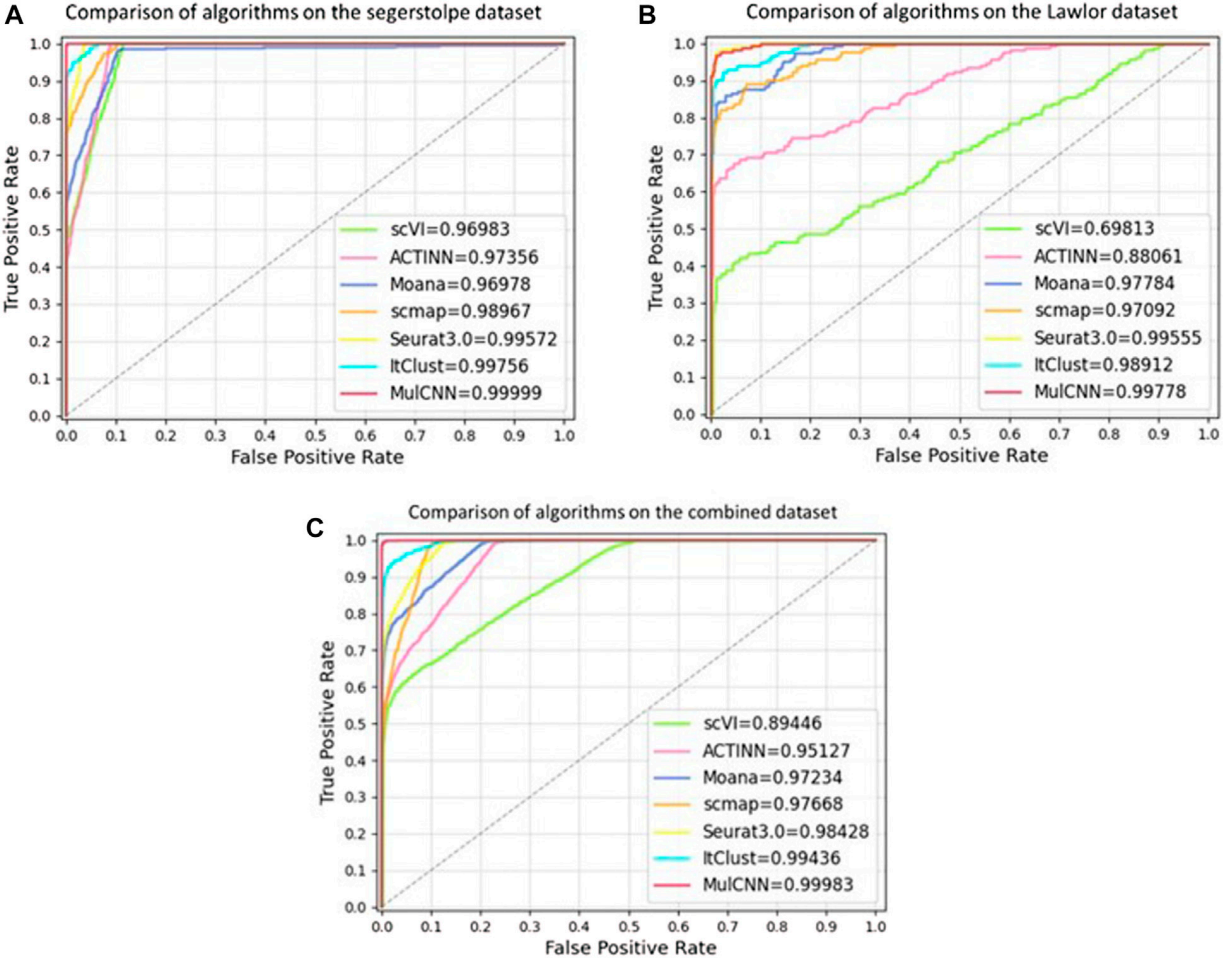
ROC curve of different models. **(A)** Comparison of ROC curves of algorithms on seger-stolpe dataset; **(B)** Comparison of ROC curves of models on Lawlor dataset; **(C)** Comparison of ROC curves of models on combined dataset.

To better evaluate the model’s performance, we plotted the ROC curves of different models in each dataset, as shown in Figure 7. The ROC curve is a composite indicator that reflects the sensitivity and specificity of continuous variables and reveals the interrelationship between sensitivity and specificity. It calculates a series of sensitivities and specificities by setting out several different critical values for the continuous variables. The area under the ROC curve is the AUC value, and the larger the area, the better the accuracy and the higher the performance. It can be observed that the curve of MulCNN is always above the other models in different datasets. These results demonstrate that the performance of our MulCNN is superior.

### 3.3 Ability to identify pseudo cell types

In many studies, the limited sample size often results in the inability to cover all cell types. As a result, supervised machine learning models are constrained by the labeled information and struggle to generate cell types outside of the training samples. When unknown cell types appear in the samples to be predicted, they are often misclassified into known cell types, which are referred to as pseudocell types. Therefore, identifying pseudocell types is crucial, and our model can avoid misclassification by labeling them as “unknown.”

**FIGURE 8:**
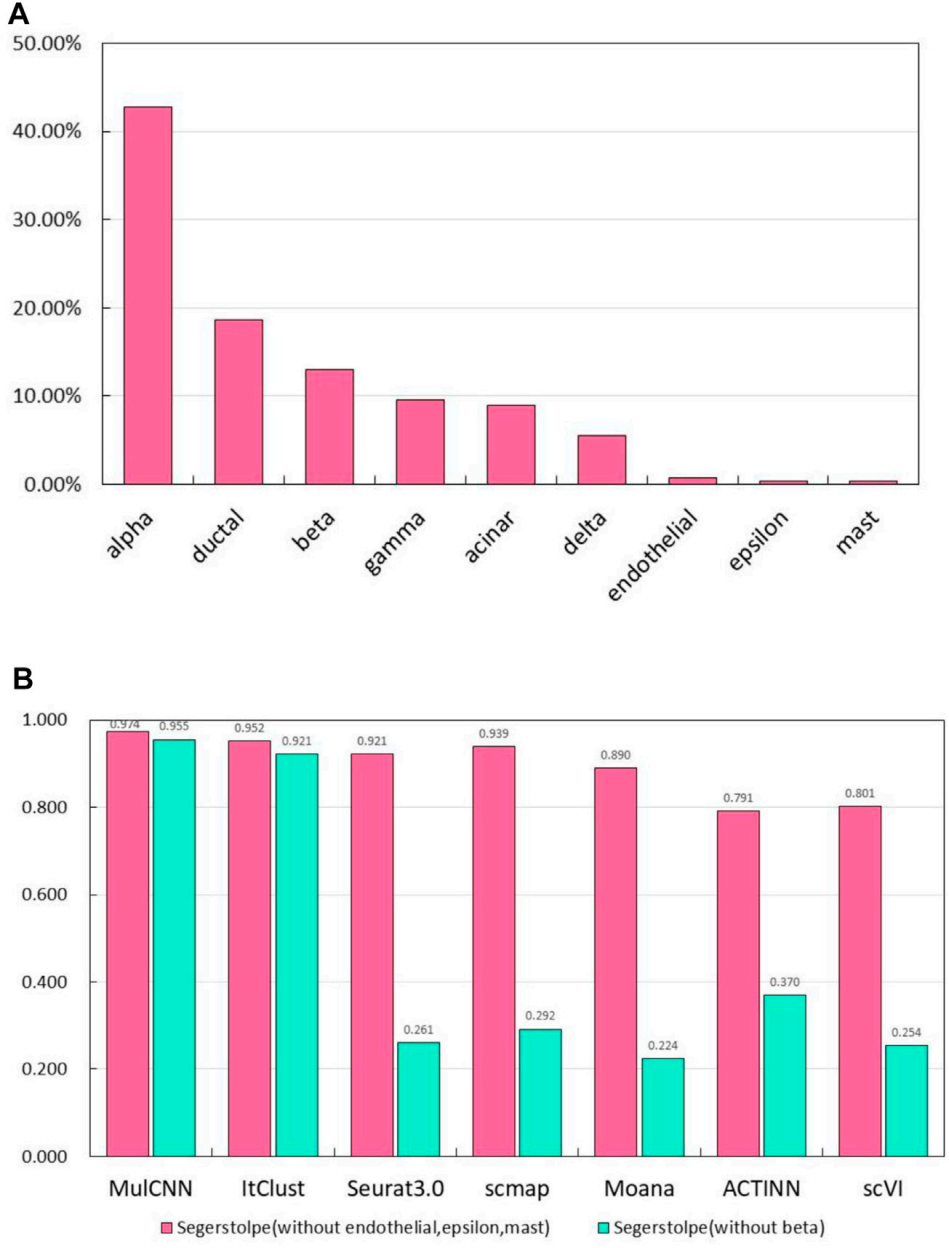
Results of experiments to identify pseudo cell types. **(A)** Per-centage of each cell type in the Segerstolpe dataset; **(B)** Accuracy of MulCNN and other super-vised algorithms in the case of missing categories in the segerstolp dataset.

To test the ability of our model to identify pseudo-cell types, we created two new training sets by removing certain cell types from the segerstolpe dataset. Specifically, we removed endothelial, epsilon, and mast cell types in one training set, and the main cell type beta in another. As shown in Figure 8A, these cell types constitute a small percentage of the segerstolpe dataset. To make the task more challenging, we evaluated the model on a test set containing all cell types. We trained the model on the two new training sets and evaluated its accuracy using a threshold of 0.97. If the model output a probability below the threshold for each cell type, it was marked as “unknown”. These experiments allowed us to investigate how the accuracy of the model for cell type classification changes when certain cell types are eliminated from the reference data.

The experimental results are presented in Figure 8B. We observed that MulCNN consistently achieved a high accuracy rate, outperforming other models, regardless of whether the primary or secondary cell types were removed from the reference dataset. Here, primary cell types refer to those with a high percentage in the dataset, while secondary cell types refer to those with a low percentage. These findings indicate that MulCNN has the ability to identify pseudo-cell types.

**FIGURE 9:**
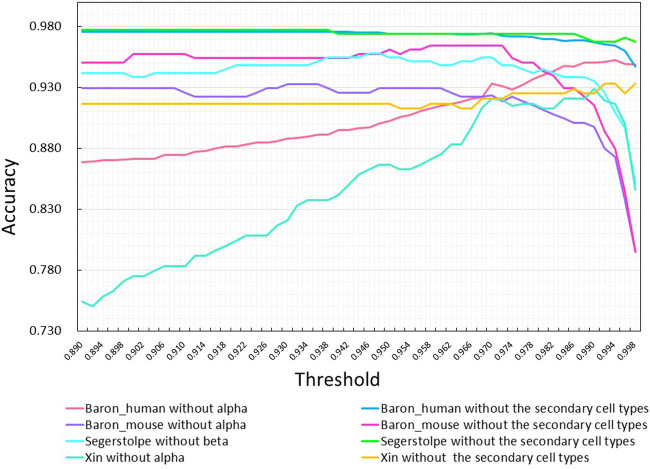
Effect of adjusting the threshold on the accuracy of MulCNN in predicting different da-tasets. Each curve corresponds to the removal of the primary or the secondary cell types from dif-fer-ent data sets.

We also studied the impact of the threshold on the classification accuracy of MulCNN. To this end, we tested various thresholds ranging from 0.89 to 0.998 with a step size of 0.002. Figure 9 shows the results of this analysis. Based on the overall performance of the model across all datasets, we found that the optimal threshold value is 0.97.

### 3.4 Ablation experiments

#### 3.4.1 Performance of models with different construction methods

To investigate the contributions and effects of different parts of the model, we conducted ablation experiments, the results of which are presented in Table 4. In particular, we evaluated the performance of our model when different parts of the convolutional pooling layer were removed. The “Without entire convolutional pooling layer” experiment involved removing the entire convolutional pooling layer from the feature extraction process and directly inputting the gene expression matrix and PCA features into the multilayer perceptron layer. The “Without partial convolutional pooling layer” experiment involved removing a part of the convolutional pooling layer (specifically, we removed “convolution and max-pooling_1” in Figure 1). According to the experimental results, all components of the model contribute to improved performance.

**TABLE 4:** Results of Ablation experiments.

Dataset	Contrast section	ACC	F1-score	ARI	Precision	Recall
Baron_human	**All**	**0.9907**	**0.8606**	**0.9855**	**0.8413**	**0.8484**
	Without entire convolutional pooling layer	0.9743	0.7008	0.9628	0.7596	0.6777
	Without the partial convolutional pooling layer	0.9891	0.7684	0.9823	0.7619	0.7768
	Without the PCA layer	0.9907	0.8404	0.9855	0.8341	0.8484
Baron_mouse	All	0.9717	0.8418	0.9864	0.8836	0.8468
	Without the entire convolutional pooling layer	0.8587	0.4304	0.7891	0.4703	0.4203
	Without the partial convolutional pooling layer	0.9717	0.7646	0.9867	0.8636	0.7278
	Without the PCA layer	0.9647	0.7283	0.9817	0.7124	0.7468
Segerstolpe	**All**	**0.9968**	**0.9949**	**0.9964**	**0.9973**	**0.9429**
	Without the entire convolutional pooling layer	0.8516	0.5238	0.7418	0.5700	0.5069
	Without the partial convolutional pooling layer	0.9935	0.9925	0.9876	0.9937	0.9916
	Without the PCA layer	0.9968	0.9949	0.9964	0.9973	0.9926
Lawlor	**All**	**0.9783**	**0.9601**	**0.9593**	**0.9918**	**0.9429**
	Without the entire convolutional pooling layer	0.6196	0.2076	0.2188	0.1983	0.2341
	Without the partial convolutional pooling layer	0.9130	0.6765	0.8632	0.6647	0.6939
	Without the PCA layer	0.9783	0.9601	0.9593	0.9918	0.9429
Xin	**All**	**0.9917**	**0.9617**	**0.9839**	**0.9949**	**0.9375**
	Without the entire convolutional pooling layer	0.8792	0.5294	0.7228	0.5693	0.5264
	Without the partial convolutional pooling layer	0.9708	0.8599	0.9501	0.9809	0.8125
	Without the PCA layer	0.9792	0.8790	0.9676	0.9856	0.8438

#### 3.4.2 Batchsize adjustment

**FIGURE 10:**
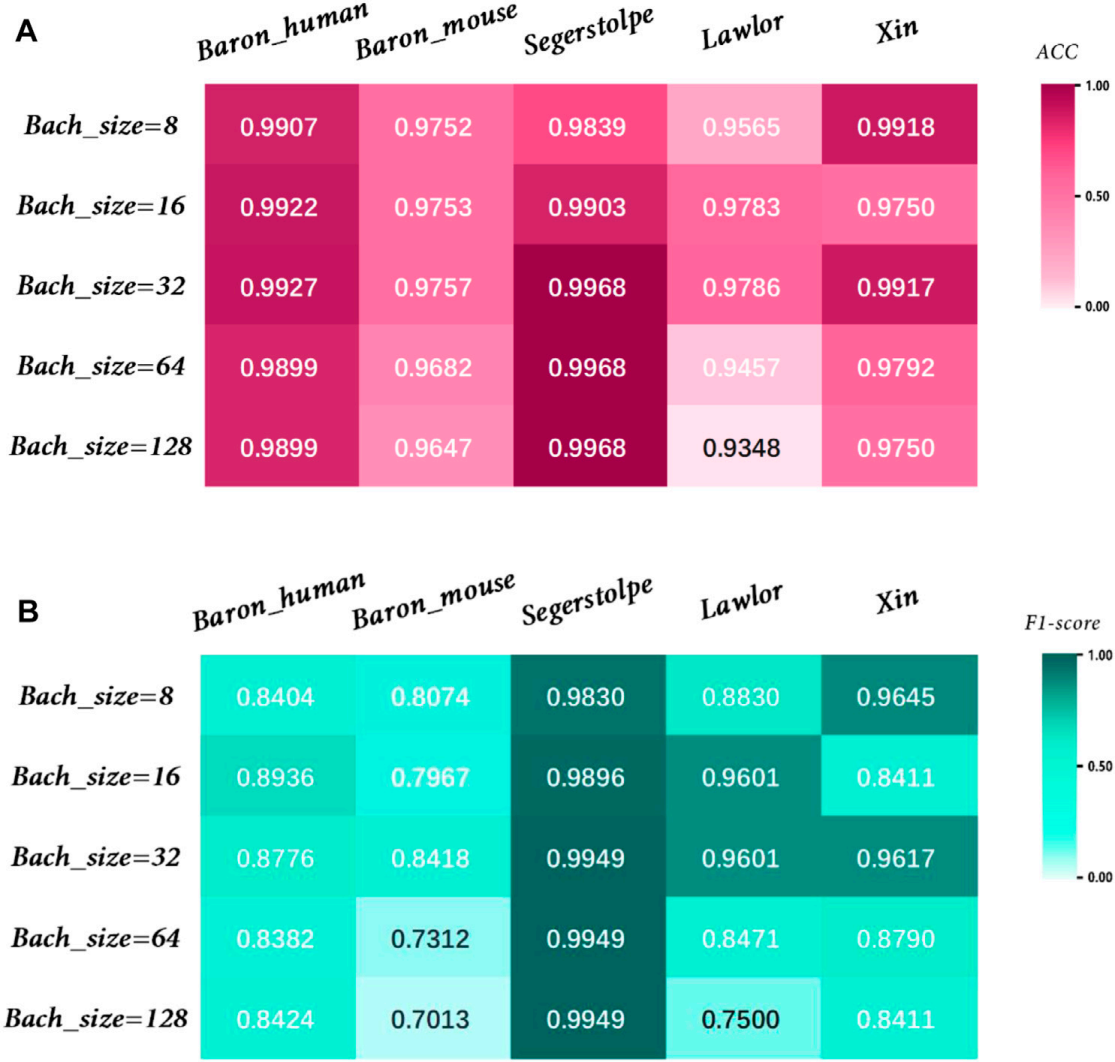
Performance analysis of MulCNN under different Bachsize. **(A)** Accuracy comparison; **(B)** F1 score comparison.

The effect of parameters on model accuracy needs to be considered, so we investigated the effect of different Batchsize values on model performance, as shown in Figure 10. We used the training set to train the model, the validation set to tune the hyperparameters of the model, and the test set to evaluate the model’s generalization capabilities. Through a comprehensive evaluation of different datasets, we found that the model achieved the best performance when Batchsize was set to 32.

## 4 Discussion

Single-cell transcriptomics is a powerful technique that can provide gene expression profiles of individual cells. However, there are several challenges associated with downstream analysis of scRNA-seq data, such as the lack of standardized dataset formats, reference gene expression profiles, high dimensionality, sparsity, and the presence of noise in the data. Deep learning techniques have shown great promise in addressing these challenges by leveraging the unique features of scRNA-seq data. MulCNN, have shown great promise in overcoming these challenges and providing accurate cell type identification. MulCNN employs a unique feature selection method to exclude genes that do not play a role in identifying cell types, which not only enhances visualization but also reduces noise and improves computational efficiency. By addressing these bottlenecks in downstream analysis, MulCNN offers a solution to better understand the complexity of scRNA-seq data and its potential implications for disease diagnosis and treatment.

## 5 Conclusion

In this study, we introduce MulCNN, a deep learning approach that utilizes multiscale convolutional neural networks to predict cell classes based on gene expression. We have evaluated MulCNN using datasets from different species that have been processed using various techniques and generated using four distinct platforms (InDrop, SMART-Seq2, Fluidigm C1, SMARTer). We compared MulCNN with other unsupervised clustering methods and found that it consistently achieves high Adjusted Rand Index (ARI) without the need to fine-tune hyperparameters such as resolution. Additionally, MulCNN outperforms popular supervised cell type classification methods such as scmap, Seurat 3.0, Moana, ItClust, ACTINN, and scVI in all evaluation scenarios.

MulCNN’s success can be attributed to its unique approach to feature extraction. The method uses multi-scale convolution to extract cell type-specific gene expression features, which enhances the features and filters out noise through the convolution operation, extracting key spatial features that enhance the classification performance of the model. Through comparison with several popular methods on publicly available datasets, we have demonstrated MulCNN’s superior performance in cell classification. Furthermore, despite the availability of many neural network-based cell classification tools, MulCNN stands out for its efficiency, lightweight design, and accuracy. MulCNN introduces a new extension direction for analysis tools of single-cell RNA-sequencing data. Its success in accurately identifying cell types in scRNA-seq data has the potential to significantly advance our understanding of cell biology and disease progression, ultimately leading to improved diagnosis and treatment methods.

## Data Availability

The original contributions presented in the study are included in the article/supplementary material, further inquiries can be directed to the corresponding author.
